# Spatio-temporal dynamics of arbuscular mycorrhizal fungi and soil organic carbon in coastal saline soil of China

**DOI:** 10.1038/s41598-020-66976-w

**Published:** 2020-06-17

**Authors:** Huan-Shi Zhang, Ming-Xi Zhou, Xue-Ming Zai, Fu-Geng Zhao, Pei Qin

**Affiliations:** 1 Nanjing Institute for Comprehensive Utilization of Wild Plants, Nanjing, 210042 China; 2 State Key Laboratory of Soil and Sustainable Agriculture, Institute of Soil Science, Chinese Academy of Sciences, Nanjing, 21008 China; 3 Key Laboratory of Soil Environment and Pollution Remediation, Institute of Soil Science, Chinese Academy of Sciences, Nanjing, 21008 China; 4 Biology Centre,Czech Academy of Sciences, Institute of Plant Molecular Biology, Ceske Budejovice, 37005 Czech Republic; 5 Horticulture Department, Jinling Institute of Technology, Nanjing, 210038 China; 6 Halophyte Research Lab, Nanjing University, Nanjing, 210093 China

**Keywords:** Wetlands ecology, Wetlands ecology, Wetlands ecology, Wetlands ecology

## Abstract

A comprehensive understanding of the relationship between arbuscular mycorrhizal (AM) fungi and coastal saline soil organic carbon (SOC) is crucial for analysis of the function of coastal wetlands in soil carbon sequestration. In a field experiment, the temporal and spatial dynamics of AM fungi, glomalin-related soil protein (GRSP) – which is described as a N-linked glycoprotein and the putative gene product of AM fungi, SOC, and soil aggregates were investigated in halophyte *Kosteletzkya virginica* rhizosphere soil of coastal saline areas of North Jiangsu, China. Soil samples were collected from a depth of up to 30 cm in two plantation regions from August 2012 to May 2013. Results showed *K. virginica* formed a strong symbiotic relationship to AM fungi. AM colonization and spore density were the highest in the 10–20 cm soil layer of Jinhai farm in August 2012, because of the presence of numerous fibrous roots in this soil layer. The total GRSP and SOC were the highest in the 0–10 cm soil layer in May 2013 and November 2012, respectively. Correlation coefficient analysis revealed that AM colonization and spore density were positively correlated with total GRSP. Meanwhile, total GRSP was significantly positively correlated with large macroaggregates (>3 mm), SOC, total P, Olsen P, and soil microbial biomass carbon (SMBC), but negatively correlated with microaggregates (<0.25 mm), soil EC, total N, and pH. SOC was positively correlated with spore density, large macroaggregates, small macroaggregates (2–0.25 mm), alkaline N, and SMBC and negatively correlated with microaggregates, EC, pH, and total K. Although it may be a statistical artifact, we found an interesting phenomenon that there was no significant correlation between soil aggregates and AM colonization or spore density. Hence, total GRSP is a vital source of saline soil C pool and an important biological indicator for evaluating coastal saline SOC pool and soil fertility, while AM colonization or spore density may not be.

## Introduction

A lot of research shows hydrological and biogeochemical processes are essential for material and energy exchange among climate-soil-plant systems, and understanding the spatiotemporal features of the water and carbon (C) cycles is of great importance for watershed ecosystem management^1,2^. Coastal wetlands are one of the most productive ecological systems worldwide and are located in the transition zones of marine and terrestrial ecosystems^3^. Soil is the core of coastal wetland C budget and the largest organic C pool; as such, slight changes in soil will affect C emissions into the atmosphere^3^. Furthermore, the structure of soil aggregates, which are the basic unit of soil structure and the site of metabolism and transformation of matter and energy in the soil, confers physical protection against mineralization and decomposition of soil organic C (SOC). Microbial activity is limited within the aggregates, allowing 0.4 to 1.2 Pg C to be sequestered each year in soil aggregates^4,5^. Therefore, aggregate formation is considered an important soil C sequestration mechanism^6^. The stability of soil aggregates is also important to maintain soil porosity, gas exchange, erosion resistance, and water holding capacity^7^. Hammer & Rillig^8^ reported that large amounts of Na^+^ in coastal saline soil solute induce colloid formation and flocculation, leading to difficulty in the formation of colloidal aggregates. Thus far, limited information is available regarding the relation between soil aggregates and the stability of SOC pool in coastal saline land.

Soil aggregation can be altered directly by management strategies or indirectly by biotic and abiotic factors. Among biotic factors, symbiosis between arbuscular mycorrhizal (AM) fungi, an important group of beneficial microorganisms in soil, and the roots of most land plants contribute to the stability of soil aggregates^9–11^, including those in high-salinity soils, such as salt marshes^12^. AM fungi naturally occur in salt environments and promote the salinity tolerance of plants by enhancing nutrient acquisition^13–15^, decreasing Na uptake^16^, and alleviating water stress^17^. The large network of AM external mycelium can entangle soil organic and inorganic particles^18^ and thus promote the formation of macroaggregates^9,19^. Meanwhile, hyphae release an alkaline-soluble glycoprotein compound called glomalin or glomalin-related soil protein (GRSP), which acts as a soil particle binding agent, similar to mucopolysaccharides produced by soil bacteria^20^. GRSP demonstrates 3–10 times higher soil aggregating ability than hot-water extractable carbohydrates, and its concentration in soil is strongly positively correlated with the water-stability of soil aggregates^21^. GRSP is very resistant to decomposition, having a half-life of 7–42 years^22^. It accumulates in soils to account for 4%–5% of soil C, which exceeds the 0.08%–0.2% contribution of soil C from soil microbial biomass^22^. Therefore, GRSP is linked to soil C storage via its effect on soil aggregate stabilization and is a vital component of SOC^23^.

A strong positive relationship exists between GRSP concentration and the amount of water-stable aggregates in non-saline soils^11,21,24^. Adame *et al*. found a negative relationship between GRSP and salinity (which ranged from 0.02 mg·g^−1^ to 1.38 mg·g^−1^) in mangrove estuary soils of southeast Australia^25^. Hammer & Rillig suggested that salinity can stimulate AM fungi to secrete GRSP^8^. To our knowledge, no study has directly tested the relationship among GRSP, soil aggregates, and SOC in coastal saline land.

*Kosteletzkya virginica* (L.) Presl. is a perennial dicot halophyte found along the Atlantic and Gulf coasts of the United States. It is highly tolerant to salinity^26–28^ and other abiotic stresses^29,30^. China contains about 2 × 10^7^ hm^2^ of coastal saline land, which plays an important role in soil C sequestration. In 1993, *K. virginica* was introduced into China to improve coastal tidal flats, develop saline agriculture and it has potential to make phytoremediation in coastal zone^31,32^. This species could be used to produce biofuel^33^, fodder additive^32^, natural medicine^34^, and other raw materials^34^. Planting *K. virginica* in saline soil improves soil organic matter content and promotes plant diversity in the coastal saline region of Jiangsu Province, China^32^. Under controlled greenhouse conditions, inoculation of AM fungi increased the available P and soil enzyme activities in bulk soil and improved plant growth under various salt stress conditions^35,36^. However, no information is available regarding the relationship between AM fungi in *K. virginica* rhizosphere soil and the C pool of coastal saline land.

The main objectives of this study were to evaluate AM fungal status, GRSP, soil organic carbon and soil aggregates concentrations to answer the following questions: (i) are there spatio-temporal differences in SOC, AM fungal colonization and spore density associated with *K. virginica* in coastal saline soils? And (ii) could AM fungi and GRSP be biological indicators for evaluating coastal saline SOC pools? And (iii) what are the relationships between AM fungi and soil aggregates in coastal saline soils?

## Results

### Spatial and temporal changes in AM colonization and spore density

**Figure 1:**
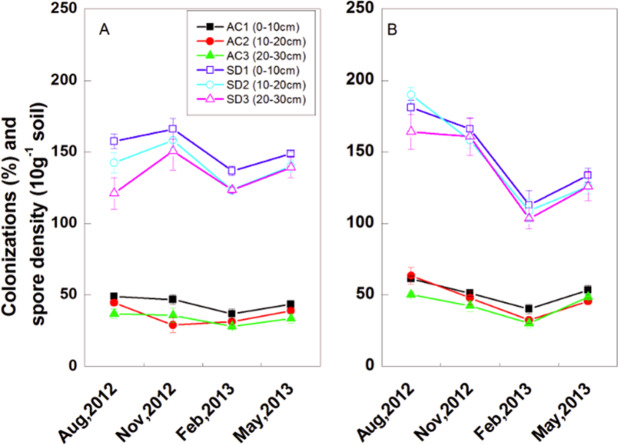
Spatio-temporal variation of AM colonization (AC) and spore density (SD) in the rhizosphere of *K. virginica* in Qingkou saltern (**A**) and Jinhai farm (**B**). AC1: AM colonization in 0–10 cm soil layer. AC2: AM colonization in 10–20 cm soil layer. AC3: AM colonization in 20–30 cm soil layer. SD1: spore density in 0–10 cm soil layer. SD2: spore density in 10–20 cm soil layer. SD1: spore density in 20–30 cm soil layer. Data are means ± SE of five replicates. Comparisons among means were made with the Least Significant Difference (LSD) test.

Data on AM colonization and spore density in control plot were not presented because no AM fungi were found. In Qingkou saltern and Jinhai farm plantations, the AM colonization rate and spore density decreased with increasing soil depth (Fig. 1A,B). The AM colonization rate and spore density in the 10–20 cm soil layer were higher than those in the 0–10 cm soil layer of Jinhai farm plantation in August 2012 (Fig. 1B. *p* > 0.05). In Qingkou saltern plantation, AM colonization rate in 10–20 cm soil layer was lower than that in 20–30 cm soil layer in November 2012 (Fig. 1A). In Qingkou saltern and Jinhai farm, the AM colonization rate decreased from the highest value at August 2012 to the lowest value at February 2013. The spore density in Qingkou saltern showed a similar trend, that is, the highest spore density in the 0–10 cm soil layer was recorded in November 2012.

### Spatial and temporal distribution of total GRSP

**Figure 2:**
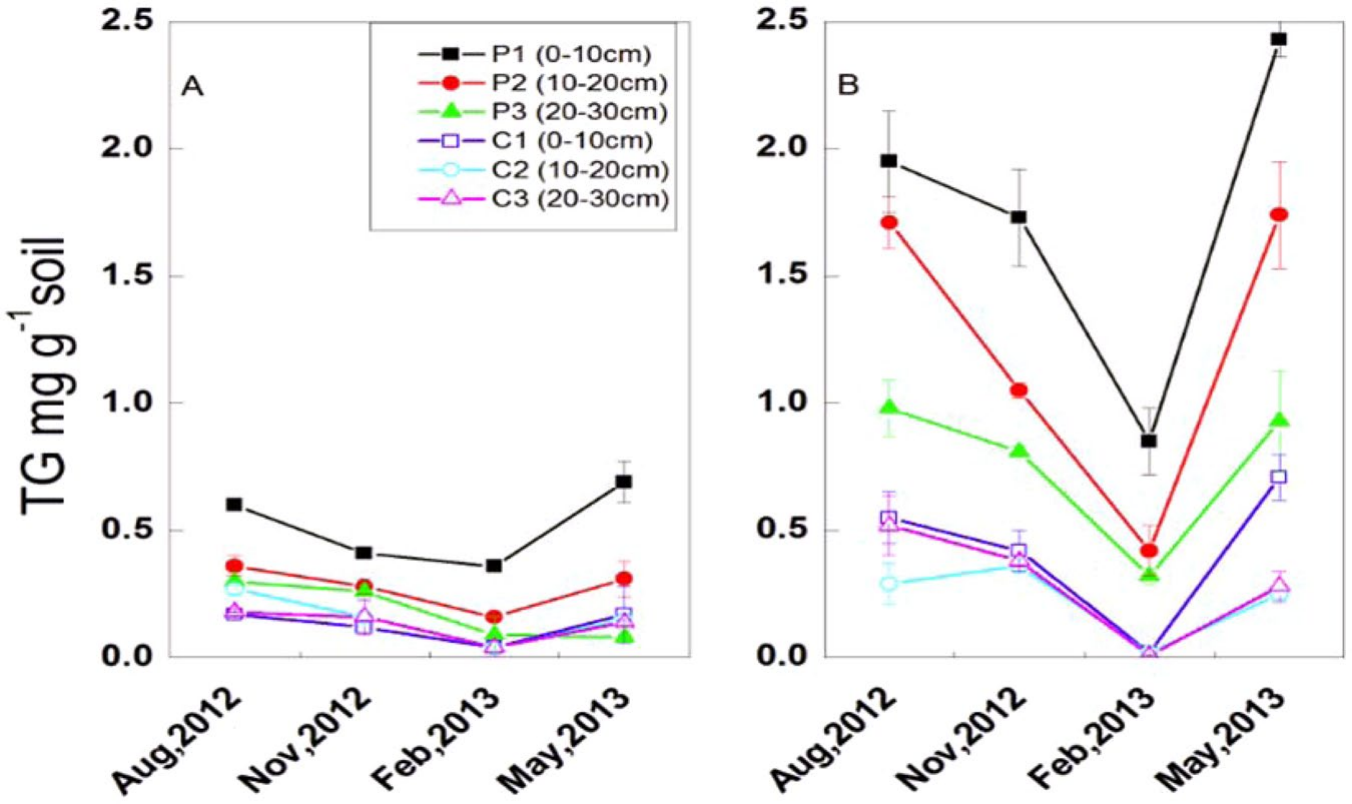
Spatio-temporal distribution of total GRSP (TG) of plantations and control plot in Qingkou saltern(**A**) and Jinhai farm (**B**). P1: 0–10 cm soil layer in *K. virginica* plantation. P2: 10–20 cm soil layer in *K. virginica* plantation. P3: 20–30 cm soil layer in *K. virginica* plantation. C1: 0–10 cm soil layer in control plot. C2: 10–20 cm soil layer in control plot. C3: 20–30 cm soil layer in control plot. Data are means ± SE of five replicates. Comparisons among means were made with the Least Significant Difference (LSD) test.

In Qingkou saltern and Jinhai farm, the total GRSP content decreased with increasing soil depth and ranged from 0.08 mg·g^−1^ to 0.69 mg·g^−1^ and 0.32 mg·g^−1^ to 2.43 mg·g^−1^, respectively (Fig. 2A,B). The total GRSP content in *K. virginica* plantation is significantly higher in Qingkou saltern than that in control plot in the same layer (*p* < 0.05). Irrespective of soil layer, the total GRSP content decreased from August 2012 to the lowest values in February 2013 and then returned to high levels in May 2013.

### Edaphic factors

**Figure 3:**
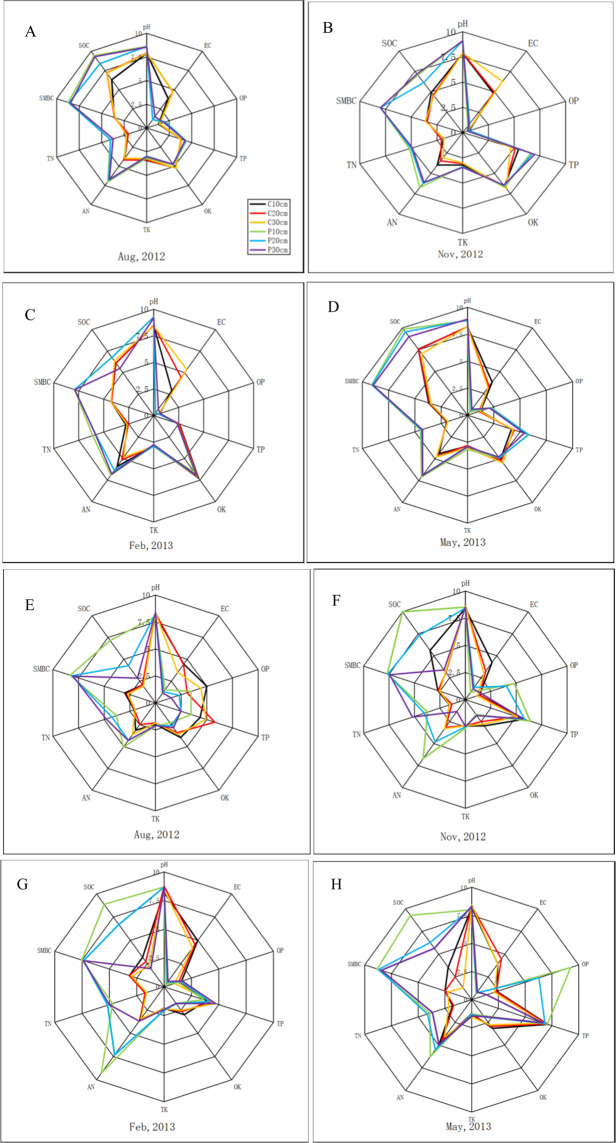
Spatio-temporal variation of edaphic factors of *K. virginica* plantations and control plot in Qingkou saltern (**A**–**D**) and Jinhai farm (**E**–**G**). After data processing, all factors values range from 0 to10. Different color and style of lines represented different soil layers. C10cm: 0–10 cm soil layer in control plot. C20cm: 10–20 cm soil layer in control plot. C30cm: 20–30 cm soil layer in control plot. P10cm: 0–10 cm soil layer in *K. virginica* plantation. P20cm: 10–20 cm soil layer in *K. virginica* plantation. P30cm: 20–30 cm soil layer in *K. virginica* plantation. Data are means ± SE of five replicates. Comparisons among means were made with the Least Significant Difference (LSD) test.

Ten edaphic factors were assessed to comprehensively determine the local soil condition. The results were presented by a radar chart. The plantation soil pH, available nitrogen, total nitrogen, and soil microbial biomass carbon (SMBC) are all significantly higher in Qingkou saltern than in control plot from August 2012 to May 2013 (*p* < 0.05, Fig. 3A–D). The SOC content ranged from 6.34 mg·g^−1^ to 14.2 mg·g^−1^ in the plantation. The plantation SOC in the 20–30 cm soil layer is significantly higher than that in the 10–20 cm soil layer in August and November 2012 and significantly lower than that in control plot soil in February 2013 (*p* < 0.05). The electrical conductivity (EC) in all plantations is significantly lower than that in control plot soils in each soil layer (*p* < 0.05). The EC increased in the plantation with increasing depths of the soil layer. In August 2012 and May 2013, the Olsen phosphorus of the plantation is significantly higher than that of control plot in each soil layer (*p* < 0.05). The total phosphorus of the plantation is significantly higher than that of control plot (*p* = 0.024), except for that in February 2013. However, no significant difference was observed between K content in the plantation and control plot in each soil layer (*p* > 0.05).

In Jinhai farm, soil pH was not significantly different between the plantation and control plot from August 2012 to May 2013 (Fig. 3E–H). The EC and Olsen potassium in the plantation are significantly lower than those in control plot in each soil layer (*p* < 0.05). By contrast, the total nitrogen, SMBC, and SOC in the plantation are significantly higher than those in control plot in each soil layer in four measurements (*p* < 0.05), except for SOC in the 20–30 cm soil layer during November 2012 and February 2013. The SOC content ranged from 2.69 mg·g^−1^ to 10.71 mg·g^−1^ in the plantation. The Olsen phosphorus in the plantation is significantly higher than that in control plot in each soil layer during November 2012 and May 2013 (*p* < 0.05).

### Spatial and temporal distribution of soil aggregates

**Figure 4:**
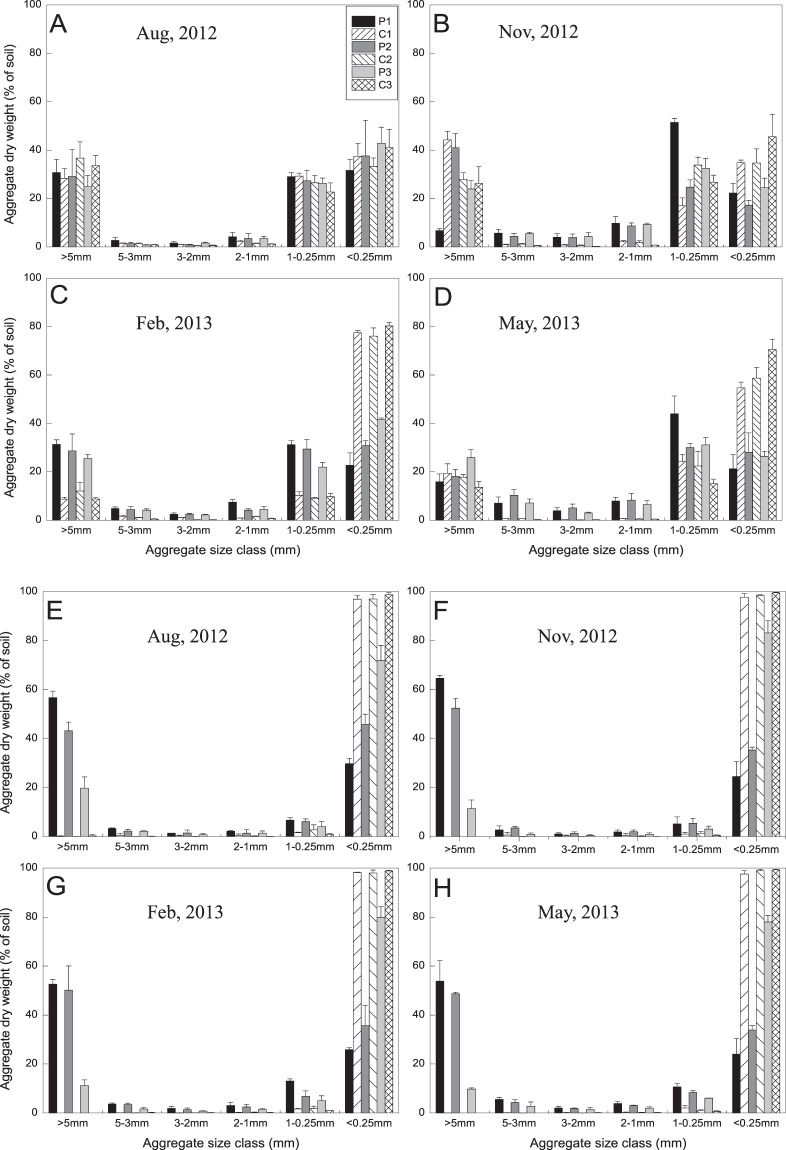
Aggregates-size distribution as determined by wet sieving for the 0–10 cm, 10–20 cm and 20–30 cm layers in Qingkou saltern (**A–D**) and Jinhai farm (**E–H**). P1: 0–10 cm layer in *K. virginica* plantation. P2: 10–20 cm soil layer in *K. virginica* plantation. P3: 20–30 cm soil layer in *K. virginica* plantation. C1: 0–10 cm soil layer in control plot. C2: 10–20 cm soil layer in control plot. C3: 20–30 cm soil layer in control plot. Data are means ± SE of five replicates. Comparisons among means were made with the Least Significant Difference (LSD) test.

In Qingkou saltern, irrespective of plantation or control plot in the four periods, soil aggregates are mainly in the fractions of > 5 mm, 1–0.25 mm, and microaggregates (MI) (Fig. 4A–D). In August 2012, all soil aggregate fractions between the plantation and control plot exhibited no significant difference. In November 2012, the soil aggregate contents of 3–5, 2–3, and 1–2 mm fractions in the plantation are significantly higher than those in control plot in the three soil layers (*p* < 0.05). However, the content of > 5 mm fraction in the plantation is significantly lower than that in control plot in the 0–10 cm soil layer (*p* = 0.026). The large macroaggregates (LM) and small macroaggregates (SM) contents in the plantation are significantly higher with time than those in control plot in February 2013, but the MI content in the plantation is significantly lower than that in control plot in the three soil layers. In May 2013, the soil aggregate contents in 3–5, 2–3, and 1–2 mm fractions are higher than those in control plot, and the MI is significantly lower than that in control plot in each soil layer.

In Jinhai farm, the results of the parameters tested are similar among the four periods (Fig. 4E–H). In the plantation, soil aggregates are mainly in > 5 mm and MI. In control plot, soil aggregates are mainly in MI in the three soil layers during the four periods. In all soil layers and periods, the contents of LM and SM in the plantation are significantly higher in Jinhai farm than those in control plot, and the MI content in control plot is significantly higher than that in Jinhai farm in the plantation (*p* = 0.031).

### Correlation coefficient analysis

AM colonization was negatively correlated with pH, EC, total nitrogen, and Olsen potassium (*p* < 0.01) and positively correlated with Olsen phosphorus (*p* = 0.038), SMBC, spore density, and total GRSP (*p* < 0.01, Table 1). Spore density was negatively correlated with pH, EC, and total nitrogen (*p* < 0.01) and positively correlated with SOC, total potassium (*p* < 0.05), and total GRSP (*p* = 0.005). Total GRSP was also positively correlated with LM, SOC, total phosphorus, Olsen phosphorus, and SMBC but negatively correlated with MI, EC, total nitrogen, and pH (*p* < 0.01). SOC was positively correlated with LM, SM, available nitrogen (*p* < 0.01), and SMBC (*p* = 0.033) and negatively correlated with MI, EC, pH (*p* < 0.01), and total potassium (*p* = 0.047). LM content was positively correlated with SM, SOC, available nitrogen, and total GRSP (*p* < 0.01) and negatively correlated with MI (*p* = 0.007) and total nitrogen (*p* = 0.036). SM content was positively correlated with SOC (*p* = 0.005), available nitrogen, and SMBC (*p* < 0.05) and negatively correlated with MI, EC, total potassium (*p* < 0.01), and OK (*p* = 0.038). MI was negatively correlated with SOC, available nitrogen, and total GRSP (*p* < 0.01). Finally, no significant correlation was observed between soil aggregates and AM colonization or spore density (*p* > 0.05).

**Table 1:** Correlation matrix of soil aggregates, edaphic factors, AM fungus, and soil total GRSP in Jinhai farm.

	LM	SM	MI	EC	pH	SOC	TN	TP	TK	OP	AN	OK	SMBC	SD	AC	TG
LM	1															
SM	0.473^**^	1														
MI	−0.986^**^	−0.615^**^	1													
EC	−0.038	−0.460^**^	0.122	1												
pH	−0.316	−0.150	0.312	−0.438^**^	1											
SOC	0.544^**^	0.741^**^	−0.629^**^	−0.481^**^	−0.338^*^	1										
TN	−0.334^*^	−0.223	0.342^*^	−0.310	0.610^**^	−0.188	1									
TP	0.098	0.145	−0.116	0.020	−0.224	0.011	−0.320	1								
TK	0.048	−0.543^**^	0.060	0.344^*^	0.269	−0.544^**^	0.127	−0.306	1							
OP	0.189	0.208	−0.209	0.055	−0.393^*^	0.257	−0.496^**^	0.721^**^	−0.473^**^	1						
AN	0.447^**^	0.408^*^	−0.478^**^	−0.504^**^	0.256	0.431^**^	0.086	−0.202	0.126	−0.091	1					
OK	−0.128	−0.338^*^	0.179	0.496^**^	−0.373^*^	−0.266	−0.080	−0.268	0.045	−0.190	−0.258	1				
SMBC	0.171	0.353^*^	−0.221	0.137	−0.683^**^	0.395^*^	−0.534^**^	0.543^**^	−0.708^**^	0.710^**^	−0.224	0.192	1			
SD	0.217	−0.294	−0.139	−0.558^**^	−0.616^**^	0.402^*^	−0.458^**^	0.052	0.372^*^	0.016	−0.264	0.063	0.183	1		
AC	0.322	0.038	−0.296	−0.493^**^	−0.809^**^	0.074	−0.683^**^	0.251	−0.012	0.345^*^	−0.180	−0.429^**^	0.566^**^	0.819^**^	1	
TG	0.566^**^	0.323	−0.568^**^	−0.585^**^	−0.791^**^	0.383^**^	−0.606^**^	0.490^**^	−0.290	0.625^**^	−0.059	0.145	0.729^**^	0.519^**^	0.772^**^	1

Note: Pearson product-moment correlations on the means (n = 108) are shown. * and ** indicate significant correlation at 0.05 and 0.01 probability levels, respectively.

TN: total nitrogen, TP: total phosphorus, TK: total potassium, OP: Olsen phosphorus, AN: available nitrogen, OK: Olsen potassium, SD: spore density, AC: AM colonization, TG: total GRSP, the same as below.

## Discussion

### AM fungus and edaphic factors

The inoculation of AM fungi can form good symbiotic association with *K. virginica* roots under various salt stress conditions in greenhouse^35,36^. This study demonstrated that *K. virginica* in the coastal saline soil of North Jiangsu could also form symbiotic relationships to AM fungi. He *et al*. reported that the maximal value of the AM colonization and spore density occurred at the 0-10 cm soil layer in farming-pastoral zone^37^. In this study, the AM colonization rate and spore density in the 10–20 cm soil layer are higher than those in the 0–10 cm soil layer because of the presence of numerous fibrous roots in the former. For the same reason, *K. virginica* possessed numerous fibrous roots during August 2012, and the highest AM colonization rate was detected during this period. AM fungal hyphae in roots can be related to the absorption and translocation of low-mobility nutrients, such as P, in soil and water from distant areas that are inaccessible to plant roots^38^. In the present study, increased hyphal colonization could help in efficient nutrient and water absorption and transportation from soil to host, leading to increased nutrient demands in August. This result is in accordance with the reports that the maximum abundance of AM fungi occurs during summer and the colonization declines during winter and early spring^39–41^. However, Füzy *et al*. reported that AM colonization peaked in late spring to early summer and exhibited a second peak later in autumn^42^. This may be due to different soil and air temperature changes and plant growth characteristics leading to the difference of AM colonization.

In this study, the AM colonization and spore density in the two research sites decreased with increasing soil depth (Fig. 1A,B), consistent with the results of He *et al*.^37,43^, Taniguchi *et al*.^44^, Wang *et al*.^45^, and Zhang *et al*.^41^. This phenomenon might be due to the usual distribution of the main roots of *K. virginica* in 0–20 cm soil. Moreover, the EC and pH increased but the oxygen concentration decreased with increasing soil depth (Fig. 3A–G). The correlation coefficient analysis results also showed significantly negative correlation of AM colonization and spore density with pH and EC (Table 1). These results corroborate reports on reduction in root mycorrhizal rate at high salinity levels^35,36,46–49^. This may be due to salinity be able to hamper colonization capacity, spore germination, and growth of AM fungal hyphae^46,50,51^. Füzy *et al*. suggested that drought may play an important role in governing mycorrhizal activity in saline habitats because AM fungi may help plants acquire water from soil^42^; in addition, the formation of additional arbuscules may facilitate the transfer of water and nutrients to plants. Although mycorrhizal colonization is reduced with increasing salt levels, the symbiosis between AM fungi and halophytes may be strengthened in saline environments once the partnership has been established^52^.

Bencherif *et al*. found that the number of AM fungal spores increased with soil salinity level^49^. Hildebrandt *et al*.^53^ and Becerra *et al*.^54^ also reported different halophytic plants associated with numerous AM fungus spore populations in saline soils. In addition, Aliasgharzad *et al*.^13^ reported that salt stress can stimulate AM fungi sporulation. Bencherif *et al*.^49^ suggested that sporulation can be considered a resistance behavior to help AM fungi survive adverse environmental conditions. In contrast to these findings, the present results indicated a significant reduction in spore count as salinity level increased. This phenomenon could be explained by the inhibitory nature of high salinity levels on spore-producing hypha^55^. Zhang *et al*.^41^ suggested that the high spore number found in October coincides with the end of growth season and can also reflect the accumulation of spores throughout the growing season. In the present study, the highest spore density was observed in Qingkou saltern and Jinhai farm during November and August (Fig. 1), respectively. This finding could be due to the effects of the differences in soil type, edaphic factors, and climate characteristics in the two research areas on spore density and hyphal growth; thus, the number of spores might be different in various types of soil^56^.

In contrast to previous research^41,57^, the present work showed that seashore saline soil total nitrogen was negatively correlated with AM colonization and spore density. This finding could be due to the higher amounts of N that accumulated in mycorrhizal plants than in non-mycorrhizal plants^47^. With AM fungi colonization, *K. virginica* mycorrhizal became stronger and intensively absorbed nitrogen from saline soil, leading to a shortage of supply of N in this period. Additionally, the entire soil microbial community became more active with the improvement in soil physico-chemical properties and soil fertility, resulting in significantly intensified ammonification and nitrification and accelerated organic nitrogen mineralization. In the present study, the available P content in plantation soils is higher than that in control plot and was positively correlated with AM colonization. This trend might be explained by the fact that enhanced AM colonization can increase the available P content^36^. Magallon-Servín *et al*.^58^ suggested that AM can produce numerous organic acids, which can solubilize mineral P. For example, the metabolic activity of the *K. virginica* root system enhances the reproduction of AM fungi and other soil microorganisms, thereby accelerating the release of soluble phosphatase into the soil^36^.

### AM fungus and GRSP in coastal saline soil

AM colonization is positively correlated with GRSP content^41,59–63^. Consistent with previous works, the present study reported that AM colonization, similar to spore density, was positively correlated with GRSP, illustrating the presence of the majority of GRSP in AM fungal hyphae and spores^64^. Similar to the findings of He *et al*.^43^ and Taniguchi *et al*.^44^, the current results showed that the total GRSP content in each plantation decreased with soil depth. In Qingkou saltern, the average total GRSP contents in the 30 cm soil layer are 35.44% and 62.5% lower than those in the 20 and 10 cm soil layers, respectively. In Jinhai farm, the average total GRSP contents in the 30 cm soil layer are 33.98% and 56.75% lower than those in the 20 and 10 cm soil layers, respectively. These findings may be due to the distribution depth of the *K. virginica* root. The usual distribution depth of the mature *K. virginica* roots is 20 cm, which supports the result of Taniguchi *et al*.^44^ and confirms that the reduction of root biomass may cause the decrease in AM fungi colonization and GRSP content with soil depth. The microbial activity was strong in highly fertile soil in the 0–20 cm layer, especially at 10 cm, and favored microbial reproduction expansion and GRSP accumulation. Thus, GRSP content was concentrated in the 0–20 cm soil layer, especially in 0–10 cm.

GRSP is a very stable biomolecule with a half-life of 6–42 years in soil^22,63^, but concentrations can fluctuate throughout the growing season^65^. Moreover, inoculation of AM fungi stimulated the synthesis of easily extractable GRSP (EE-GRSP), a fraction of soil GRSP^11^, which is newly produced by the hyphae and spores of AM fungi^62^. In the present study, the highest concentrations of total GRSP in saline soil were observed in May or August, and the lowest was detected in February. These results are consistent with those reported in previous studies^43,65^, which indicated that the highest total GRSP content was observed in May. Zhang *et al*.^41^ reported that both total GRSP and EE-GRSP contents were the highest in August and lowest in October. This finding may be due to the corresponding mycorrhizal infection in different sampling periods. The local area has more suitable temperature and water and light conditions in May and August, and the plants are in a vigorous growth period. In addition, the accumulation of total GRSP decreased possibly due to the decomposition of the labile part of GRSP (EE-GRSP). Hence, the total GRSP content fluctuated seasonally because AM fungi and *K. virginica* were affected by seasonal changes, demonstrating time heterogeneity.

Different kinds of soil texture exhibit varied influences on total GRSP content. In agricultural, natural grassland, desert, and mangrove forest ecosystems, the total GRSP contents are 1–21 mg·g^−1^, 4.5–5.0 mg·g^−1^, 2.49–4.11 mg·g^−1^, and 0.46–1.38 mg·g^−1^, respectively^21,25,41,59^. In the present study, the total GRSP content in the *K. virginica* plantation in Jinhai farm is 0.85 m·g^−1^ to 2.43 mg·g^−1^, which is lower than that in natural grassland soil but higher than that in the mangrove forest ecosystem. This finding indicates that *K. virginica* could establish good symbiotic association with AMF^35,36^ and contribute to the restoration of saline soil. The total GRSP content is higher in Jinhai farm than in Qingkou saltern (Fig. 2) because of the heavy clay soil texture in the latter and the sandy loam in the former. AM fungi obtained more suitable soil conditions for growth in the Jinhai farm soil, thus demonstrating higher AM colonization and spore density in August, 2012 (Fig. 1B). Furthermore, GRSP concentration may be influenced by soil mineral and fertility. In the current study, total GRSP showed a significantly positive relationship to SOC, total phosphorus, Olsen phosphorus, and SMBC, consistent with the findings of many researches^22,41,43,57,66^. This result could be due to the requirement of mycorrhizal fungal growth and metabolism^67^. Rillig reported that total GRSP is an important part of soil N^59^; in addition, many studies indicated that GRSP content is positively correlated with available nitrogen^41,43,57^. In the present study, total GRSP was significantly correlated with available nitrogen and negatively correlated with soil total nitrogen. This can be explained by former results that there is a higher portion of available nitrogen in the plantation plots.

The amount of GRSP was negatively correlated with soil EC and pH. Our results are strongly supported by other authors, who reported the significant negative effects of soil salinity on GRSP^48,68,69^. However, Ji *et al*.^59^ and Lovelock *et al*.^70^ found that GRSP levels in soil and *in vitro* cultures were negatively correlated with hyphal length, suggesting that the production of this compound may be a stress response. Hammer and Rillig^8^ reported that the GRSP content increased up to a threshold level of 150 mM NaCl but decreased with further increase in the salt concentration. Garcia *et al*. reports that maximum salinity stress (2.0 dS m^−1^) increased 6% and 18% GRSP production than 1.0 dS m^−1^ and 0.6 dS m^−1^, respectively^71^. Hence, GRSP may be involved in the inducible stress responses of AM fungi to salinity.

### GRSP and soil aggregates in coastal saline soil

The distribution patterns of soil aggregates differed in the two sites. In Qingkou saltern, both in plantation and control plot, soil aggregates in all soil layers are mainly in > 5 mm, 1–0.25 mm, and MI. In Jinhai farm, soil aggregates are mainly in > 5 mm and MI in the plantation, and 95% are MI in control plot. This finding could be directly due to difference in soil texture in the studied areas; that is, Qingkou saltern has heavy clay with high soil bulk density and low nutrition, whereas Jinhai farm has sandy loam and up to 65.50% sand (Table 2). Soil in these two sites showed very poor granulation structure. Such results suggest that soil aggregation decreased as levels of sand and carbonate increased, likely due to concurrent decreases in levels of clay in the soil^4^. In addition, the contents of macroaggregates (>0.25 mm) effectively increased in the plantation soil compared with that in CK soil. Macroaggregates were negatively correlated with microaggregates (<0.25 mm), suggesting that the former was gradually formed from the latter during planting *K. virginica* periods. This finding may be related to amount of fibrous roots can wind up dispersion soil particles and form large aggregates^72^. Furthermore, the stability of soil aggregates is probably affected more by the direct and indirect actions of the plant–fungal system, rather than by plant root metabolism^73^. In present study, SM content was positively correlated with SMBC, and LM content was positively correlated with total GRSP; as such, microorganisms are one of the most active and important biological factors.

**Table 2:** Physical and chemical properties of soils at two sites.

	Qingkou saltern	Jinhai farm
pH	8.22	8.43
Electrical conductivity (ds·m^−1^)	4.13	3.21
Soil salinity(‰)	15.35	10.16
Soil organic carbon (‰)	4.52	4.97
Total nitrogen (g·kg^−1^)	0.16	0.49
Total phosphorus (g·kg^−1^)	0.45	0.80
Total potassium (g·kg^−1^)	30.22	18.21
alkaline nitrogen (mg·kg^−1^)	10.72	39.42
Olsen phosphorus (mg·kg^−1^)	5.52	2.19
Olsen potassium (mg·kg^−1^)	552.07	212.50
Sand (%)	15.50	65.50
Silt (%)	22.50	13.50
Clay (%)	61.50	20.50

Wright *et al*.^74^ indicated that glomalin is an insoluble glue-like substance only released by AM fungi into the soil environment during hyphal turnover and after the death of the fungus^64^; this compound may contribute to binding within microaggregates and macroaggregates. Wright and Anderson^75^ found that aggregate stability and GRSP were linearly correlated (r = 0.73, n = 54, *p* < 0.001) across all treatments from different cropping systems. In relation to this finding, the present results indicated the positive correlation between LM and TG, with coefficient of 0.566 (*p* < 0.01), and the negative correlation between MI and total GRSP, with coefficient of 0.568 (*p* < 0.01). Hence, soil GSRP presents strong cementing ability, inducing the formation of aggregates with increased structural stability^63,76^. In addition, external hyphae can promote the formation of soil aggregates by physical tangles and changing soil dry–wet circulation^9,77^. Bearden and Petersen^19^ suggested that mycorrhizae primarily influence the stability of macroaggregates. By contrast, no significant correlation was found between soil aggregate and AM colonization or spore density (*p* > 0.05) in the present study. This may be due to the formation of soil aggregates takes a long time, but the fibrous roots used to check AM colonization have a shorter time to grow from the main and lateral roots of *K. virginica*. Meanwhile, it may also be affected by the detection methods and environmental factors, and there is a large gap between the test results and the actual situation of AM colonization or spore density. Rillig *et al*.^59^ reported that the direct effect of GRSP on aggregate stability is higher than the total (direct and indirect) effect of hyphae on soil aggregate stability in oak-hickory (Quercus-Carya) forests of eastern North America; this phenomenon also can partly explain the weak correlation between soil aggregate and AM fungi. Barto *et al*.^78^ suggested that abiotic factors (mowing, grazing, and fertilization) can be more important for determining soil aggregation than biotic factors (root length and mass, AM colonization, extraradical AMF hyphal length), especially in highly aggregated soils. Hence, contrary to He *et al*.’s^43^ research conclusion that spore density, colonisation of hyphae, the contents of glomalin can be used as parameters to monitor the development of organic carbon dynamic and nutrition cycle in sand soil, AM colonization or spore density may not be used to evaluate coastal saline SOC pool and soil fertility. Furthermore, we cannot ignore another possibility that the lack of correlation between soil aggregate and AM fungi was a statistical artifact. The mechanism underlying this phenomenon has not been fully understood yet. The results also showed that SM content was negatively correlated with soil EC, similar to the report of Lax *et al*.^79^. This probably due to large amount of Na ions complicate the formation of colloidal aggregates in coastal saline soil^8^.

### AM fungus and SOC in coastal saline soil

Coastal soil ecosystems dominated by plants play a critical role in the global sequestration of C^80^, and the SOC pool acts as a crucial regulator of C fluxes between biosphere and atmosphere. Our previous studies revealed that plant biomass improved by AM fungi inoculation might be beneficial to coastal soil ecosystems in sequestering large amounts of C^81^. We also found that AM fungi inoculation could strongly promote plant dry biomass and nutrient uptake by *K. virginica*, regardless of salinity level^36,81^. In the present work, after planting *K. virginica* for 3 years, the SOC in the plantation is significantly higher than that in control plot in each soil layer in several periods (*p* < 0.05). This finding could be due to the ability of *K. virginica* to sequester SOC within numerous living biomass aboveground and belowground, litter, and dead wood^81^. Moreover, the mycorrhizal roots of *K. virginica* can provide AM fungi with photosynthetic C, which, in turn, is delivered to soil via fungal hyphae^38^. This finding is confirmed by the positive correlation between spore density and SOC (*p* < 0.05) (Table 1). Rillig^22^ stated that the C in GRSP largely contributes to the total soil C pool. In tropical soils, the amount of C in GRSP is estimated to be 37%, which represents 3% of soil C pools^67^. The total GRSP-to-SOC ratio of sand and soil is between 34.88% and 66.85%^41^. In the present study, total GRSP was positively correlated with SOC (*p* < 0.05), with the highest total GRSP-to-SOC ratio of up to 53.29% in Jinhai farm. Hence, total GRSP is a vital source of saline soil C pool, and important biological indicator for evaluating coastal saline SOC pool and soil fertility.

In addition, as insoluble glue, GRSP can stabilize soil aggregates and significantly reduce organic matter degradation by protecting labile compounds within soil aggregates^75^. In agreement with these findings, the present study showed that LM was positively correlated with SOC and total GRSP (*p* < 0.01). Meanwhile, the negative correlation of MI content with SOC and total GRSP (*p* < 0.01) illustrates the importance of soil aggregate stabilization in inhibiting the degradation of organic matter and promoting soil C sequestration^4–6^. In our previous study, the introduced microbe (*Glomus mosseae*, and a phosphate-solubilizing fungus *Mortierella* sp.) can collaborate with indigenous microorganisms to promote the humification of organic materials^81^. Plant–AM fungi mutualism can improve the reestablishment of vegetation in bare saline-alkaline soil and drives the vegetation restoration to a community dominated by the original species^81,82^. In the present work, the significant positive relationship among AM colonization, total GRSP, SMBC, and SOC suggests that AM fungi play a crucial role in stimulating the growth of indigenous microorganisms, enhancing the stability of saline SOC pool and soil fertility, and promoting the reestablishment of vegetation^41^ when *K. virginica* was introduced into the coastal saline soil of North Jiangsu and other sites in China.

The SOC contents in the 20–30 cm soil layer of the two plantations intensively fluctuated following spatio-temporal dynamics and are even lower than that in control plot. These results suggest that SOC in the 20–30 cm soil layer of *K. virginica* plantation demonstrated instability and spatio-temporal heterogeneity in coastal saline soil. However, further research is required to understand the relative mechanisms.

## Conclusion

A strong symbiotic relationship was found between *K. virginica* and AM fungi after their introduction into the coastal saline soil of North Jiangsu for 3 years. This study demonstrated highly variable temporal and spatial patterns in the dynamics of AM fungi, total GRSP, and SOC, which were affected negatively by soil salinity. The significantly positive relation among AM fungi, total GRSP, and SOC were also confirmed. The results also revealed that soil aggregate stabilization, especially soil large macroaggregates (>3 mm), is crucial to maintain the stability of total GRSP and saline SOC pool. Although it may be a statistical artifact, a new phenomenon that no significant correlation exists between soil aggregate and AM fungi was observed, which contradicts previous findings. Hence, total GRSP is a vital source of saline soil C pool and an important biological indicator for evaluating coastal saline SOC pool and soil fertility, while AM colonization or spore density may not be. Future research must investigate the relation between AM fungi and saline soil aggregate to improve understanding of their roles in coastal ecosystems.

## Materials and Methods

### Study site

North Jiangsu experiences a typical temperate and monsoonal climate with four clearly distinct seasons. The study sites selected are Qingkou saltern of Lianyungang City (34°45′N, 119°11′E) and Jinhai farm of Yancheng City (32°59′N, 120°46′E), which are located in North Jiangsu and possess the highest annual average temperatures of 25.8 °C and 28.1 °C, respectively, in July and the lowest annual average temperatures of 0.7 °C and 2.0 °C, respectively, in January. The annual average precipitations of Lianyungang and Yancheng are 896.7 and 1020.5 mm, respectively, with 50% of the precipitation occurring from June to September. The soil types of Qingkou saltern and Jinhai farm are coastal meadow saline soil, and the soil textures are heavy clay and sandy loam, respectively (Table 2).

### Field and experimental sampling

At each study site, the 1200 m^2^ area without vegetation for high salt content was divided into six parts. The experimental design was full factorial, three parts were randomly selected as *K. virginica* plantation, and three *K. virginica* seeds were planted in one hole according to 0.5 m × 0.5 m design on May 4, 2009. 10 d after germination, germinants were thinned from 3 to 1 in each hole and each part had 800 plants. The residual three parts were selected as control plot without *K. virginica*. After seedling thinning, *K. virginica* was allowed to grow unmanaged from 2009 onwards. And five sites in each part were randomly selected for soil sampling, which started on August 15, 2012 and every three months thereafter until May 25, 2013. Soil samples (*n* = 5) were collected from section at depths of 0–10, 10–20, and 20–30 cm. Prior to sample collection, the upper layer of soil (approximately 5 mm) was scraped off to remove litter. The collected soil samples were stored in sealed polyethylene bags placed in an insulated container and transported to a laboratory. The soil samples (50 g) for soil microbial biomass C analyses were dried at room temperature, passed through a 4 mm sieve, and stored in sealed plastic bags at 4 °C until analysis. The remaining samples were divided into two parts after air drying. One part (100 g) was milled to obtain particles with size < 2 mm for determination of physico-chemical properties, and the other part (350 g) was used for water stability structure analysis.

### Analyses of total GRSP

Total GRSP was extracted from 1 g of soil by using the method described by Wright and Upadhyaya^21^. Extraction was conducted with 8 ml of 50 mM Na citrate (pH 8.0) at autoclave cycles of 121 °C for 60 min until the supernatant showed no red brown color typical of GRSP. Based on our previous experiment, two to three cycles are recommended. The fractions were determined through Bradford assay using bovine serum albumin as standard.

### Analyses of AM fungus spore and colonization

Soil samples (25 g each) were used to determine spore density of AM fungus. Spore number was determined by wet sieving in a 40 µm mesh and decanting, followed by sucrose density centrifugation. The suspension was carefully decanted and added with 40% sucrose solution. AM fungus spores were counted under a stereoscopic microscope at 40 ×. Sporocarps were dissected with forceps, and the released spores were counted. Spore density was expressed as number of spores per 10 g of dry soil.

Fresh roots were cut into 0.5–1.0 cm segments and washed until free of soil. The roots were then stained with 0.5% (w/v) acid fuchsin solution according to the method described by Phillips & Hayman^83^ and Zhao & He^84^. AM colonization was quantified by glass slide method, where 50 randomly selected 1 cm root segment units were microscopically examined^85^. Total colonization was expressed as the percentage of root segments colonized for a root sample.

### Analyses of soil aggregates

Soil aggregates were fractionated using a wet-sieving procedure^86,87^. After capillary wetting of 100 g of air-dried soil to field capacity, the samples were immersed in water on a nest of 5, 3, 2, 1, and 0.25 mm sieves and shaken vertically at 3 cm height for 50 times during a 2 min period. This wet-sieving procedure resulted in fractions of >3 mm LM, 2–0.25 mm SM, and <0.25 mm MI. Soil aggregates retained on each sieve were backwashed into pre-weighed containers, oven dried at 50 °C for 2–3 days, and weighed.

### Analyses of general soil properties

Soil pH was analyzed in a 1:5 soil-to-water ratio. SOC was determined by dichromate oxidization^88^. EC of soil was measured with a conductivity meter (Model DDS-11A; Leizi, Shanghai, China). SMBC was analyzed by fumigation–extraction method^89^. Available nitrogen was measured using alkaline hydrolysis diffusion method^90^. Olsen potassium in soil was determined by ammonium acetate through flame photometry. Olsen phosphorus was determined by chlorostannus-reduced molybdophosphoric blue color method after extraction with 0.5 M sodium bicarbonate for 30 min^90^. total nitrogen, total phosphorus, and total potassium concentrations of soils were determined using the method of Olsen *et al*.^90^.

### Statistical analysis

Data were subjected to ANOVA using IBM SPSS Statistics (version 19.0; IBM Corp., Armonk, NY, USA). Differences were considered significant at *p* < 0.05. The means of main effects were compared using least significant difference test after a significant ANOVA test result. Pearson linear correlations among the parameters were evaluated with SPSS 19.0.
